# Molecular Characterization of a Dual Domain Carbonic Anhydrase From the Ctenidium of the Giant Clam, *Tridacna squamosa*, and Its Expression Levels After Light Exposure, Cellular Localization, and Possible Role in the Uptake of Exogenous Inorganic Carbon

**DOI:** 10.3389/fphys.2018.00281

**Published:** 2018-03-26

**Authors:** Clarissa Z. Y. Koh, Kum C. Hiong, Celine Y. L. Choo, Mel V. Boo, Wai P. Wong, Shit F. Chew, Mei L. Neo, Yuen K. Ip

**Affiliations:** ^1^Department of Biological Sciences, National University of Singapore, Singapore, Singapore; ^2^Natural Sciences and Science Education, National Institute of Education, Nanyang Technological University, Singapore, Singapore; ^3^St. John's Island National Marine Laboratory, National University of Singapore, Singapore, Singapore; ^4^The Tropical Marine Science Institute, National University of Singapore, Singapore, Singapore

**Keywords:** bicarbonate, carbon dioxide, calcification, *Symbiodinium*, symbiosis, tridacnid

## Abstract

A *Dual-Domain Carbonic Anhydrase* (*DDCA*) had been sequenced and characterized from the ctenidia (gills) of the giant clam, *Tridacna squamosa*, which lives in symbiosis with zooxanthellae. *DDCA* was expressed predominantly in the ctenidium. The complete cDNA coding sequence of *DDCA* from *T. squamosa* comprised 1,803 bp, encoding a protein of 601 amino acids and 66.7 kDa. The deduced DDCA sequence contained two distinct α-CA domains, each with a specific catalytic site. It had a high sequence similarity with tgCA from *Tridacna gigas*. In *T. squamosa*, the DDCA was localized apically in certain epithelial cells near the base of the ctenidial filament and the epithelial cells surrounding the tertiary water channels. Due to the presence of two transmembrane regions in the DDCA, one of the Zn^2+^-containing active sites could be located externally and the other one inside the cell. These results denote that the ctenidial DDCA was positioned to dehydrate ${\text{HCO}}_{3}^{-}$ to CO_2_ in seawater, and to hydrate the CO_2_ that had permeated the apical membrane back to ${\text{HCO}}_{3}^{-}$ in the cytoplasm. During insolation, the host clam needs to increase the uptake of inorganic carbon from the ambient seawater to benefit the symbiotic zooxanthellae; only then, can the symbionts conduct photosynthesis and share the photosynthates with the host. Indeed, the transcript and protein levels of *DDCA*/DDCA in the ctenidium of *T. squamosa* increased significantly after 6 and 12 h of exposure to light, respectively, denoting that DDCA could participate in the light-enhanced uptake and assimilation of exogenous inorganic carbon.

## Introduction

The fluted giant clam, *Tridacna squamosa*, is one of 12 species of giant clams from the family Cardiidae and subfamily Tridacninae, which inhabit the shallow, tropical seawater of the Indo-Pacific coral reefs (Lucas, 1988; Neo et al., 2017). Due to the lack of overturn, tropical waters are low in nutrients (de Goeij et al., 2013), but giant clams can attain high growth rates (Rosewater, 1965) because they live in symbiosis with dinoflagellates (Norton et al., 1992) of the genus *Symbiodinium* (Clade A, C, and D; Trench, 1987; DeBoer et al., 2012) commonly known as zooxanthellae. The symbiotic zooxanthellae reside extracellularly inside a tubular system with a primary tubule originating from the stomach of the host clam. The primary tubule splits into smaller secondary and tertiary tubules that permeate mainly the extensible, fleshy and colorful outer mantle (Norton et al., 1992; Yellowlees et al., 1993). During insolation, the symbionts undergo photosynthesis and transfer some photosynthates to the host clam (Muscatine, 1990), which can satisfy ~100% of the host's energy requirements (Fisher et al., 1985; Klumpp et al., 1992). Hence, the availability of light critically affects the zooxanthellae-giant clam association, especially the growth of the host clam (Crawford et al., 1988).

While the host clam benefits from receiving photosynthates from the symbiotic zooxanthellae (Streamer et al., 1993), the symbionts require a supply of inorganic carbon (C_i_) from the host in order to support ribulose-1,5-bisphosphate carboxylase/oxygenase (RuBisCO)-catalyzed photosynthesis (Furla et al., 2005). As metabolic carbon dioxide (CO_2_) produced by the host is not sufficient to support the maximal rate of photosynthesis (Yellowlees et al., 1993), zooxanthellae residing extracellularly in the tubular system must gain access to the C_i_ present in the ambient seawater through its host. Additionally, giant clams can undergo light-enhanced calcification to increase the rate of shell-formation, which requires C_i_ as a substrate (Yellowlees et al., 1993). Hence, the host clam must absorb exogenous C_i_, presumably through its two ctenidia. A ctenidium (gill) is basically a respiratory organ with a large surface area for gas exchange, ion transport and acid-base balance in some mollusks. It is the site of light-enhanced ammonia absorption and assimilation (Hiong et al., 2017a), as well as light-dependent H^+^ excretion (Hiong et al., 2017b), in *T. squamosa*.

C_i_ in seawater is present mainly as bicarbonate (${\text{HCO}}_{3}^{-}$) and to a much lesser extent as dissolved CO_2_. These two forms of C_i_ can interconvert according to the equation CO_2_ + H_2_O ⇋ H_2_CO_3_ ⇋ ${\text{HCO}}_{3}^{-}$ + H^+^. The hydration of CO_2_ proceeds at a moderate pace in the absence of a catalyst, with a rate constant of 0.15 s^−1^. However, the dehydration of H_2_CO_3_ is relatively rapid and has a rate constant of 50 s^−1^ (Maren, 1967). This results in an equilibrium constant of *K* = 5.4 × 10^−5^ and a ratio of 340:1 for [CO_2_] to [H_2_CO_3_]. Notwithstanding the moderate rate of CO_2_ hydration and the high rate of H_2_CO_3_ dehydration without a catalyst, almost all organisms possess carbonate anhydrases (CAs; EC 4.2.1.1), which are zinc-containing enzymes catalyzing these reactions with dramatic increases in the rate of CO_2_ hydration (Supuran, 2008). CAs are needed because CO_2_ hydration and ${\text{HCO}}_{3}^{-}$ dehydration are commonly coupled to rapid physiological and biochemical processes; in particular, ${\text{HCO}}_{3}^{-}$ is associated with many transport processes. There are four genetically distinct families (α, β, γ, and δ) of CAs, and the largest and the most ubiquitous family is α-CA (Chegwidden et al., 2000). As ${\text{HCO}}_{3}^{-}$ in seawater cannot freely permeate biomembranes, it has to be absorbed through specific ${\text{HCO}}_{3}^{-}$ transporters. Alternatively, ${\text{HCO}}_{3}^{-}$ can be converted to CO_2_ which can permeate biomembranes with or without the involvement of specific channels (Nakhoul et al., 1998), but the dehydration of ${\text{HCO}}_{3}^{-}$ requires a supply of H^+^. As expected, the ctenidium of *T. squamosa* expresses a Na^+^/H^+^ exchanger 3 (NHE3)-like transporter which excretes H^+^ in exchange for Na^+^ and displays light-enhanced gene and protein expression (Hiong et al., 2017b). However, even in the presence of H^+^, the un-catalyzed reaction of ${\text{HCO}}_{3}^{-}$ dehydration is a slow process and therefore requires the participation of CAs in the ambient seawater. In fact, there are secretory types of CA (Aizawa and Miyachi, 1986; Badger and Price, 1994; Suzuki et al., 1994), but CAs secreted freely into seawater would be lost to the environment. Therefore, the ctenidium of *T. squamosa* should preferably express a type of secretory CA which is anchored to or partially embedded in the apical membrane of the epithelium in contact with the external medium. Such a CA would catalyze the formation of CO_2_ from ${\text{HCO}}_{3}^{-}$ in the ambient seawater in close proximity to the epithelial surface, facilitating its absorption. Inside the ctenidial epithelial cells, the absorbed CO_2_ must be converted back to ${\text{HCO}}_{3}^{-}$, presumably catalyzed by a cytosolic CA, in order to maintain a favorable *P*CO_2_ gradient for continuous CO_2_ uptake.

Indeed, Yellowlees et al. (1993) demonstrated that the ctenidium of *Tridacna derasa* contained a high CA activity, which could be essential to regulating the C_i_ fluxes between the seawater and the hemolymph (blood). Then, Baillie and Yellowlees (1998) purified CAs from the host tissue of *T. gigas*, and identified two CA isoforms from the ctenidium and the mantle. The larger isoform (70 kDa; tgCA) was localized to the ciliated branchial filaments and cells lining the tertiary water channels in the ctenidium, in support of a role in C_i_ absorption. Subsequently, Leggat et al. (2002, 2005) reported that the uncommon 70 kDa membrane-bound tgCA contained two separate α-CA domains. It has been proposed that tgCA plays an important role in the acquisition of exogenous C_i_ by the giant clam, and in facilitating C_i_ movement within its tissues and organs (Leggat et al., 2002; Yellowlees et al., 2008). However, the advantages of having two α-CA domains in the 70 kDa CA of *T. gigas* and their specific functions remain enigmatic. Notably, symbiotic cnidarians, including corals and anemones, are not known to express CAs with dual domains.

To test the hypothesis that, like *T. gigas, T. squamosa* also expressed a membrane-bound CA with two α-CA domains, this study was undertaken to clone, sequence, and characterize a Dual-Domain CA (*DDCA*/DDCA) from its ctenidium. Particularly, efforts were made to analyze whether the DDCA comprised any transmembrane region (TM), which might shed light on the relative location of the two catalytic domains with reference to the membrane. Furthermore, the gene expression of *DDCA* in various tissues and organs were examined to test the hypothesis that the ctenidium was the main site of *DDCA* expression. Efforts were also made to determine the effects of 3, 6, or 12 h of light exposure on the transcript level of *DDCA* in the ctenidium, as compared with controls kept in darkness for 12 h. In order to elucidate whether the molecular changes observed, if any, were related to circadian rhythm, the effects of exposure to 12 h + 12 h (a total of 24 h) of darkness were also examined. Furthermore, a polyclonal anti-DDCA antibody was developed commercially to quantify the change in protein abundance of DDCA in the ctenidium in response to light exposure, and to elucidate the cellular and subcellular localization of DDCA in the ctenidium through immunofluorescence microscopy. It was hypothesized that the DDCA had an apical localization in the ctenidial epithelia, and its gene and protein expression levels could be up-regulated by light exposure. Results obtained were expected to provide insights into how DDCA, with its dual catalytic domains, could function to augment C_i_ uptake and assimilation in the ctenidium of *T. squamosa* during insolation.

## Materials and methods

### Human and animal rights

No institutional (National University of Singapore Institutional Animal Care and Use Committee) approval is required for invertebrates including giant clams at the time the laboratory experiments were performed. The animals were anaesthetized with 0.2% phenoxyethanol before killing to minimize stress and suffering.

### Animals

Adult specimens of *T. squamosa* (521 ± 184 g; mean ± SD) were procured from Xanh Tuoi Tropical Fish, Ltd (Vietnam) and kept in an indoor aquarium. Giant clams (*N* = 28) were maintained in a glass tank with recirculating seawater. The water conditions and light intensity were provided as described previously (Ip et al., 2015). Experiments were conducted after the animals were acclimatized to laboratory conditions for 1 month.

### Experimental conditions and tissue collection

For molecular work, four giant clams were killed for tissue sampling at the end of a 12 h dark period, and they were regarded as controls (*N* = 4). Another 12 individuals were exposed to light and sampled after 3, 6, or 12 h of light exposure (*N* = 4 for each time point). In order to elucidate if there was any circadian effect, four individuals were exposed to a total of 24 h of darkness, which acted as parallel controls to those exposed to 12 h of light, notwithstanding that giant clams would never encounter more than ~12 h of darkness in nature. The anaesthetized clams were dissected along the pallial line to obtain samples of the colorful and fleshy outer mantle. Thereafter, the whitish inner mantle, ctenidium, hepatopancreas, foot muscle, byssal retractor muscle, kidney and heart were quickly excised, blotted dry and immediately freeze-clamped with aluminum tongs pre-cooled by liquid nitrogen. The frozen samples were stored in −80°C until processing. For immunofluorescence microscopy, the ctenidium samples were collected from another 8 giant clams, 4 of which were exposed to darkness for 12 h and another 4 exposed to light for 12 h (*N* = 4 each).

### Total RNA extraction and cDNA synthesis

The extraction and purification of total RNA from the tissues of *T. squamosa* were performed using the TRI Reagent™ (Sigma-Aldrich, St. Louis, MO, USA) and the RNeasy Plus Mini Kit (Qiagen, Hilden, Germany), respectively. A Shimadzu BioSpec-nano spectrophotometer (Shimadzu, Kyoto, Japan) was used to quantify the purified RNA, and its integrity was determined by electrophoresis. Four micrograms of purified RNA were then used to synthesize the first strand cDNA using a RevertAid™ first strand cDNA synthesis kit (Thermo Fisher Scientific, Waltham, MA, USA).

### Polymerase chain reaction (PCR), cloning and RACE-PCR

The partial *DDCA* sequence was obtained using primers (Table 1) designed according to the conserved regions of *T. gigas tgCA* (AY790884.1) and *Phreagena okutanii* two domain membrane-associated *CA* (LC007965.1). The primer set was designed in such a way that they could differentiate against the two *CA* sequences with double domains from the algae, *Dunaliella salina* (U53811.1) and *Porphyridium purpureum* (D86051.1). PCR, cloning and sequencing procedures were performed according to the methods in Hiong et al. (2017a,b). Analyses of multiple clones of *DDCA* fragments did not reveal the presence of isoforms. The complete coding sequence of *DDCA* was obtained using a 5′ and 3′ RACE kit (SMARTer RACE cDNA amplification kit; Clontech Laboratories, Mountain View, CA, USA) and specific primers (Table 1). The cDNA sequence of *DDCA* has been deposited into GenBank with the accession number MF084997.

**Table 1:** Primers used for polymerase chain reaction (PCR), rapid amplification of cDNA ends (RACE) PCR, gene expression in tissues/organs, and quantitative real-time PCR (qPCR) of the *Dual Domain Carbonic Anhydrase* (*DDCA*) and α*-Tubulin* from the ctenidium of *Tridacna squamosa*.

**Gene**	**Primer type**	**Primer sequence (5′−3′)**
*DDCA*	PCR	Forward: CAAAGATGTCTGGAGGTGG
		Reverse: TCCAACAACTGCTAATCC
	5′-RACE	CAGTCAGCAGGCATTGTATTGGCGAA
	3′-RACE	CACACTCACAACGGCAGGAAATACCC
	Gene expression	Forward: CTATCCATGAATTTCGCCA
		Reverse: TATTTCCTGCCGTTGTGAG
	qPCR	Forward: GATACCAACCAGCCCATCAC
		Reverse: GAAGTCGTAAGCTCTACCCTG
*α-Tubulin*	qPCR	Forward: GTGCCAAAGGATGTCAATGTC
		Reverse: CTTAGCCATATCTCCGCCTG

### Gene expression in various tissues and organs

Using specific primers (Table 1) designed for *DDCA* of *T. squamosa*, PCR was conducted on the cDNA of all tissues. The cycling conditions were 94°C (3 min), followed by 35 cycles of 94°C (30 s), 50°C (30 s), 72°C (1 min) and 1 cycle of final extension at 72°C (10 min). Products were then separated by gel electrophoresis using a 1% agarose gel.

### Deduced amino acid sequence and phenogramic analyses

The *DDCA* of *T. squamosa* was translated into the deduced amino acid sequence using the ExPASy Proteomic server (Gasteiger et al., 2003; https://web.expasy.org/translate/). The DDCA sequence obtained was then aligned and compared with the *T. gigas* CA (AAX16122.1) using BioEdit (Hall, 1999). The percentage sequence identity between DDCA of *T. squamosa* and *T. gigas* CA was then computed. The TM and signal peptide were predicted using the TOPCONS program (http://topcons.cbr.su.se/). The glycosylphosphatidylinositol (GPI) anchor was predicted using the PredGPI predictor program (http://gpcr.biocomp.unibo.it/predgpi/pred.htm). A phenogramic analysis by the neighbor-joining method with 1,000 bootstrap replicates using Phylip (Felsentein, 1989) was performed with selected amino acid sequences (mainly from human) from GenBank with the aim to confirm that DDCA of *T. squamosa* was a type of membrane-associated CA and was distinctly separated from the CAs of algae.

### qPCR

The mRNA expression levels of *DDCA* were determined using the absolute quantification method with reference to a standard curve (Gerwick et al., 2007). qPCR was performed according to the procedures as described in Hiong et al. (2017a,b) and specific primers (Table 1) for *DDCA* were used. The absolute number of transcripts was computed using the standard curve and expressed as copy numbers per ng of total RNA. Although the absolute quantification method was adopted in this study, efforts were made to confirm that the transcript level of a reference gene (α*-Tubulin*) was not light-dependent. Using a pair of specific qPCR primers (Table 1), it had been confirmed that the mRNA expression level of α*-Tubulin* remained unchanged throughout the 12 h of light exposure as compared to the control kept in darkness (results not shown).

### Sodium dodecyl sulfate-polyacrylamide (SDS-PAGE) electrophoresis and western blotting

Protein extraction and SDS-PAGE were performed following the methods of Hiong et al. (2017a,b). Twenty five micrograms of proteins from the ctenidium were electrophoretically separated and transferred to PVDF membranes. Thereafter, membranes were blocked with 5% skim milk in TTBS (pH 7.6) for 1 h at 25°C. After blocking, membranes were incubated with a custom-made DDCA antibodies (epitope: SYDGHGDTKGPSDW) developed by GenScript (Piscataway, NJ, USA) using 1:800 dilution, or anti-α-tubulin antibodies (12G10, 1:800 dilution) for 1 h at 25°C. The primary antibodies were diluted in TTBS prior to use. Subsequently, the membranes were incubated with alkaline phosphatase-conjugated secondary antibodies (Santa Cruz Biotechnology Inc.; 1:10,000 dilution) for 1 h at 25°C. A peptide competition assay was also performed to check for specificity of the anti-DDCA antibody. Visualization of bands at the predicted molecular mass was done using a BCIP/NBT Substrate Kit (Life Technologies). The bands were quantified as described in Hiong et al. (2017a,b) and the relative protein abundance of DDCA normalized with α-tubulin was reported.

### Immunofluorescence microscopy

Immunofluorescence microscopy was performed following the method of Hiong et al. (2017b). The concentrations of anti-DDCA antibody and secondary antibody Alexa Flour 488 used were 2.5 μg ml^−1^. Both primary and secondary antibodies were diluted in Pierce Fast Blocking Buffer (Thermo Fisher Scientific Inc.). A peptide competition assay was also performed to confirm the specificity of the anti-DDCA antibody following the methods of Hiong et al. (2017b). The slides were observed using Olympus BX60 epifluorescence microscope (Olympus Corporation, Tokyo, Japan) equipped with Olympus DP73 digital camera (Olympus Corporation). Figures were prepared using Adobe Photoshop CS6 (Adobe Systems, New York, USA).

### Statistical analyses

Statistical analyses were performed using the SPSS program version 21 (IBM Corporation, Armonk, NY, USA). Results were presented as mean + S.E.M. Data were evaluated for homogeneity of variances using the Levene's test. Differences between means were evaluated using the one-way analysis of variance (ANOVA), followed by multiple comparisons of means by Tukey (for equal variance) or by Dunnett's T3 (for unequal variance), and were regarded as statistically significant when *P* is smaller than 0.05.

## Results

### Nucleotide sequence, deduced amino acid sequence, and phenogramic analysis

The complete cDNA coding sequence (1,803 bp) of *DDCA* (Accession number MF084997) was obtained from the ctenidium of *T. squamosa*. It encoded for a protein of 601 amino acids with an estimated molecular mass of 66.7 kDa. The deduced DDCA sequence shared high similarity (84.3%) with the tgCA of *T. gigas* (Accession number AAX16122).

**Figure 1:**
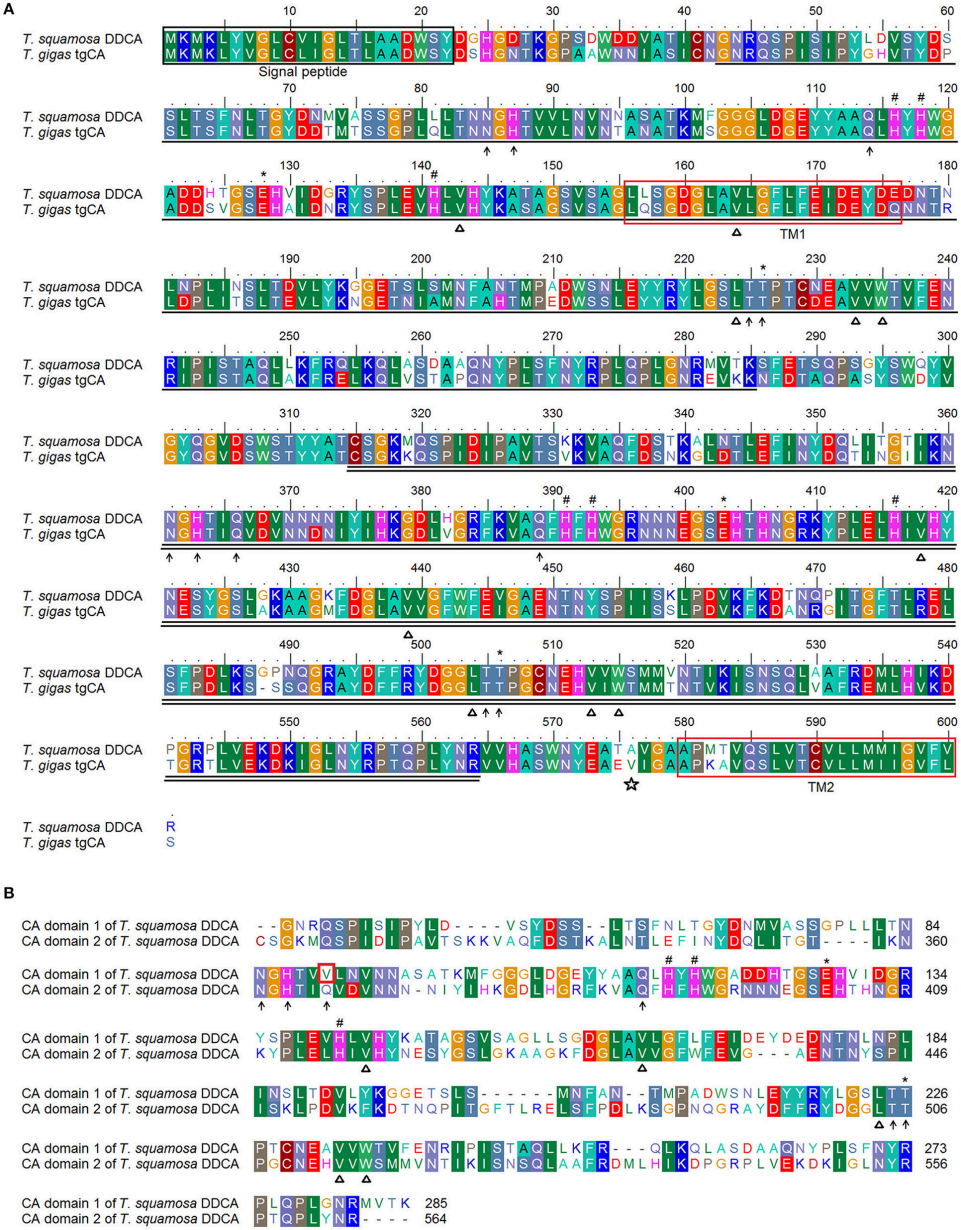
Molecular characterization of the Dual Domain Carbonic Anhydrase (DDCA) of *Tridacna squamosa*. **(A)** An amino acid sequence alignment of the DDCA of *T. squamosa* and tgCA of *T. gigas*. **(B)** A comparison of the two CA domains of the DDCA of *T. squamosa*. The shaded residues indicate identical or highly similar amino acids. The signal sequence is labeled and indicated by a black box. The residues that are single underlined make up the first CA domain, while the second CA domain is double underlined. The open star marks the putative residue which attaches a GPI anchor. The asterisks indicate the gatekeeper residues that allow efficient proton-transfer. The residues that coordinate the catalytic Zn^2+^ ion are marked by hash signs. The open triangles indicate the residues that form the CO_2_ binding sites. The hydrophilic residues that make up the binding sites for ${\text{HCO}}_{3}^{-}$ are marked by arrows. The transmembrane domains (TMs) are indicated by a red box. The lack of one hydrophilic residue that is involved in ${\text{HCO}}_{3}^{-}$ binding in the first CA domain of DDCA is marked by a blue box.

A protein blast of the deduced DDCA of *T. squamosa* revealed the presence of two α-CA domains and a signal peptide (residue 1–22; Figure 1A). The first α-CA domain (residue 43–285) bore similarities with membrane-associated CAs (CA4/CA9/CA15; Table S1) while the second α-CA domain (residue 315–564) display high similarity with CA4 (Table S2). There was a ~53% sequence similarity between these two α-CA domains. Based on the TOPCONS program (http://topcons.net/; Tsirigos et al., 2015), the DDCA of *T. squamosa* probably consisted of two TMs. The first TM (residue 156–176) was located inside the first CA domain while the second TM (residue 580–600) was located near the 3′ end of the second CA domain adjacent to the C-terminus. The signal peptide (residue 1–22) was predicted to be extracellular, and the GPI anchor was predicted to be Ala576 located near the 3′ end of the second CA domain (Figure 1A).

Each α-CA domain of the DDCA of *T. squamosa* had its own set of catalytic and active sites (Figure 1B). The first α-CA domain comprised the three histidine residues (His116, His118, His141) which coordinate the Zn^2+^-containing catalytic site, the hydrophobic residues (Val143, Val164, Leu224, Val233, Trp235) that form the CO_2_ binding pocket, and the gatekeeper residues (Glu128, Thr226). In the first α-CA domain, the active binding site for ${\text{HCO}}_{3}^{-}$ and H^+^ constituted five hydrophilic residues (Asn85, His87, Gln114, Thr225, and Thr226), of which His87 acted as a proton shuttle for CO_2_ hydration. Similarly, the Zn^2+^-containing catalytic site (His391, His393, His416), the hydrophobic residues that form the CO_2_ binding pocket (Val418, Val439, Leu504, Val513, Trp515), and the gatekeeper residues (Glu403, Thr506) were conserved in the second CA domain. Unlike the first α-CA domain, six hydrophilic residues (Asn361, His363, Gln366, Gln389, Thr505, and Thr506) were found in the second α-CA domain, and His363 served as a proton shuttle for CO_2_ hydration (Figure 1B).

**Figure 2:**
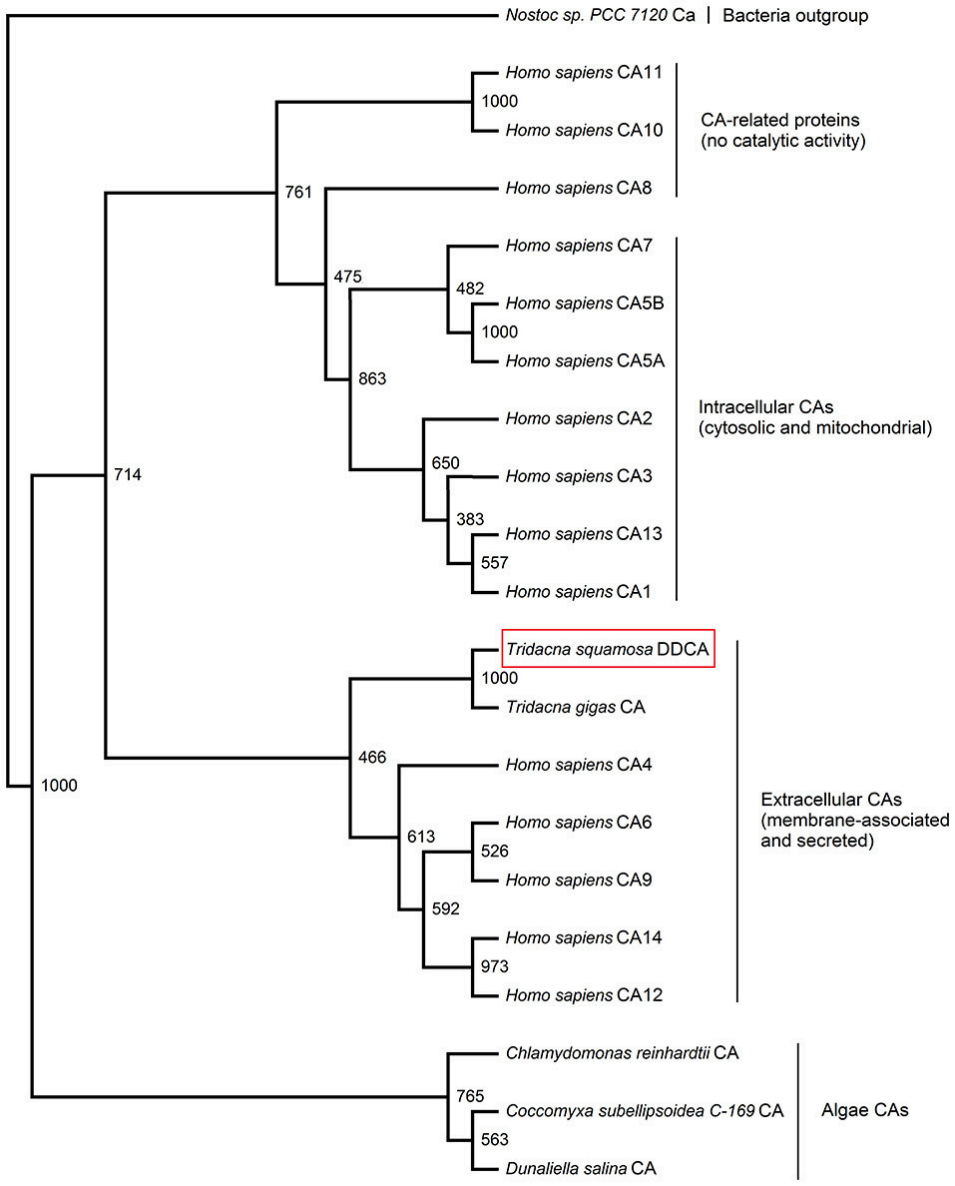
A phenogramic analysis of the Dual Domain Carbonic Anhydrase (DDCA) of *Tridacna squamosa*. A phenogram illustrating the relationship of the DDCA of *T. squamosa* with all known CAs of *Homo sapiens, Tridacna gigas* CA (tgCA) and CAs of several species of algae. The Ca of the bacterium *Nostoc* sp. (*PCC7120*) was used as the outgroup. The number located at each branch point represents the bootstrap value (max = 1,000).

A phenogramic analysis indicated that the DDCA was probably a type of extracellular (membrane-bound and/or secreted) α-CA (Figure 2). In addition, the genetic distance signified the difference between the DDCA from *T. squamosa* and selected CAs from algae, confirming that it had a host (animal) origin.

### Gene expression of *DDCA* in various organs/tissues of *T. squamosa*

**Figure 3:**
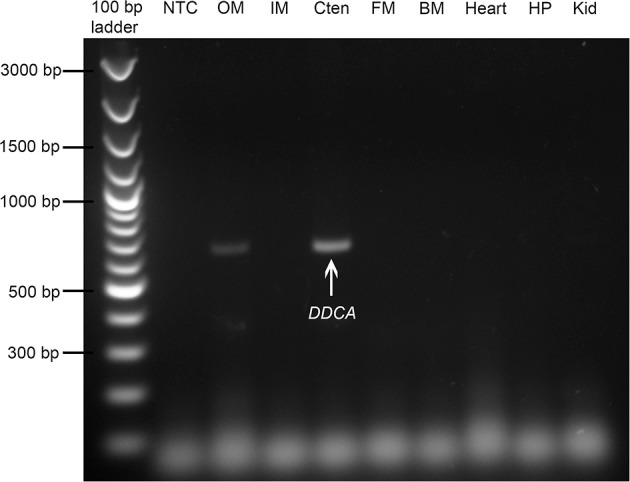
Gene expression of the *Dual Domain Carbonic Anhydrase* (*DDCA*) in tissues/organs of *Tridacna squamosa*. The mRNA expression of the *DDCA* in the outer mantle (OM), inner mantle (IM), ctenidium (Cten), foot muscle (FM), byssal muscle (BM), heart, hepatopancreas (HP), and kidney (Kid) of *T. squamosa* kept in darkness for 12 h (control). A negative control (NTC) was included in the first lane.

Among all the organs/tissues examined, the *DDCA* was expressed predominantly in the ctenidium of *T. squamosa* (Figure 3). Thus, this study focused on changes in transcript levels and protein abundance of the *DD*CA/DDCA in the ctenidium.

### Effects of light exposure on the mRNA expression level and protein abundance of *DDCA*/DDCA in the ctenidium

**Figure 4:**
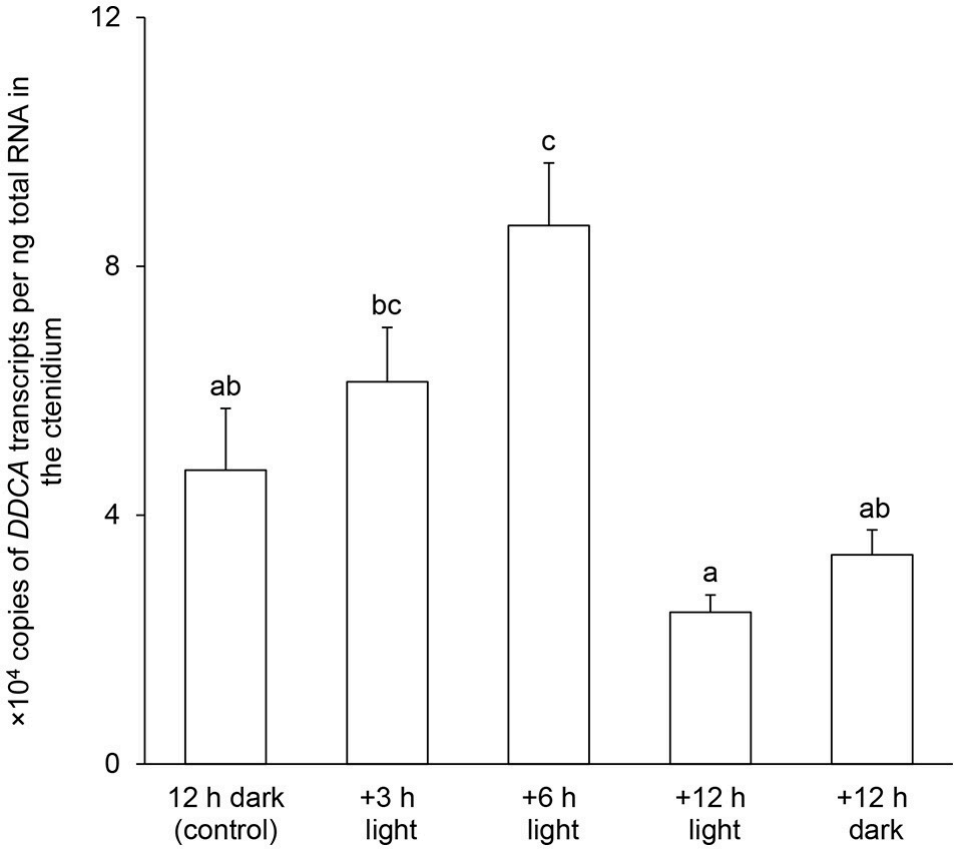
Effects of light on the mRNA expression level of *Dual Domain Carbonic Anhydrase* (*DDCA*) in the ctenidium of *Tridacna squamosa*. Absolute quantification (× 10^4^ copies of transcript per ng of total RNA) of *DDCA* transcripts in the ctenidium of *T. squamosa* kept in darkness for 12 h (control), or exposed to light for 3, 6 or 12 h, or kept in darkness for another 12 h (a total of 24 h in darkness). Results represent means + S.E.M. (*N* = 4). Means not sharing the same letters are significantly different from each other (*P* < 0.05).

**Figure 5:**
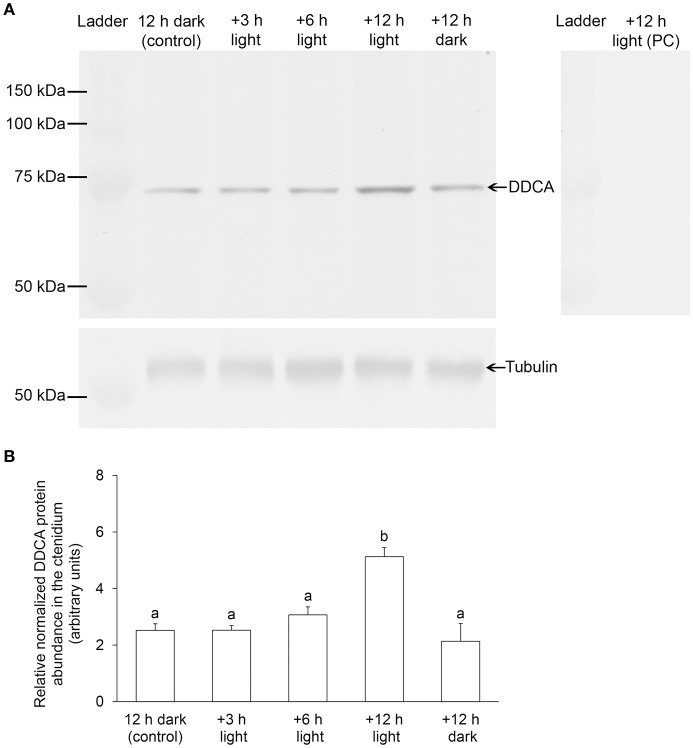
Effects of light on the protein abundance of Dual Domain Carbonic Anhydrase (DDCA) in the ctenidium of *Tridacna squamosa*. The protein abundance of DDCA in the ctenidium of *T. squamosa* kept in darkness for 12 h (control), or exposed to light for 3, 6 or 12 h, or kept in darkness for another 12 h (a total of 24 h in darkness). **(A)** Examples of an immunoblot of DDCA and tubulin (reference protein), and an immunoblot of DDCA using the anti-DDCA antibody pretreated with peptide competition (PC). **(B)** The optical density of the DDCA band for a 25 μg protein load was normalized with respect to that of tubulin. Results represent means + S.E.M. (*N* = 4). Means not sharing the same letter are significantly different from each other (*P* < 0.05).

There was a significant increase (2-fold) in the transcript level of *DDCA* in the ctenidium of *T. squamosa* exposed to light for 6 h as compared to that of the control in darkness (Figure 4). The transcript level of *DDCA* in the ctenidium of clams exposed to 12 h of light was comparable to that of the control (12 h of darkness) and the parallel control exposed to 24 h of darkness. Results from Western blotting revealed a band of interest at ~70 kDa, which was close to the estimated molecular mass of the DDCA (66.7 kDa) and therefore identified as the targeted protein (Figure 5). There was a significant increase in the protein abundance of DDCA in the ctenidium of *T. squamosa* exposed to light for 12 h as compared to the control kept in darkness for 12 h. Furthermore, the protein abundance of DDCA in the ctenidium of individuals exposed to 24 h of darkness was comparable to that of the control (12 h of darkness), and was significantly lower than that of the clams exposed to 12 h of light (Figure 5).

### Immunofluorescence microscopy

**Figure 6:**
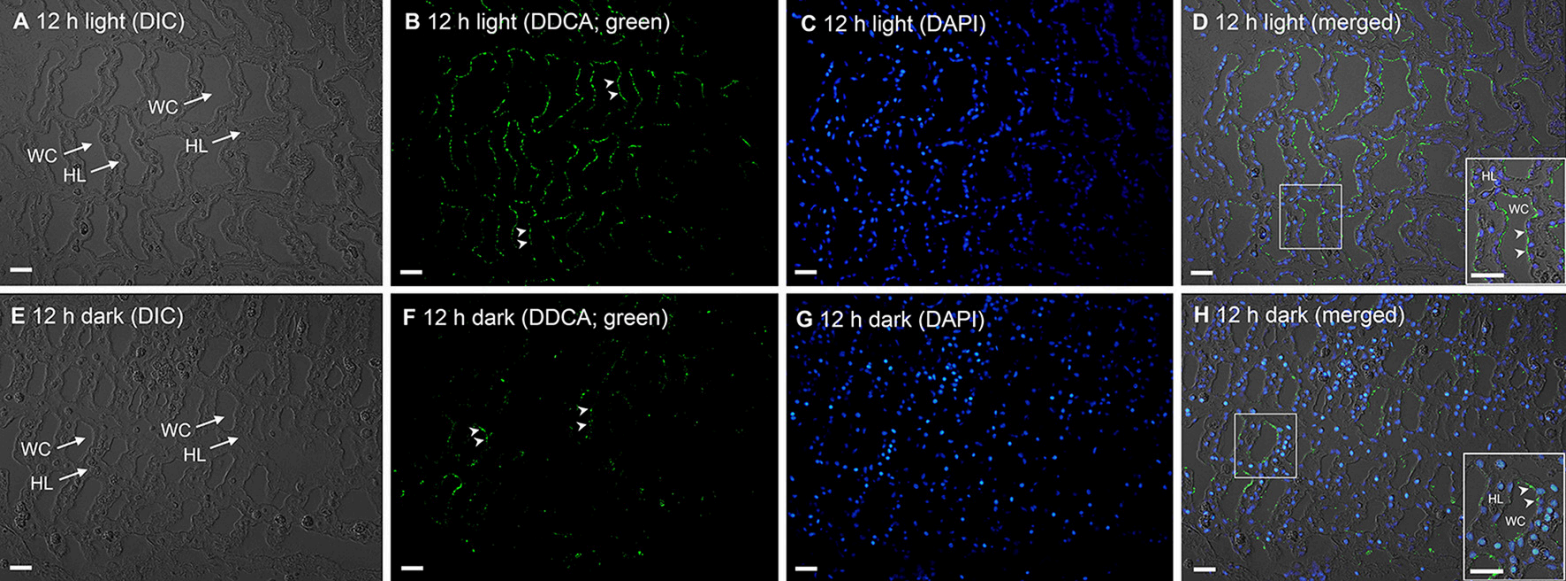
Immunofluorescence localization of the Dual Domain Carbonic Anhydrase (DDCA) in the tertiary water channels (WCs) of the ctenidium of *Tridacna squamosa*. Immunofluorescence localization of the DDCA in the WCs of the ctenidium of *T. squamosa* exposed to 12 h of light **(A–D)** or 12 h of darkness (control; **E–H**). Differential interference contrast (DIC) images show the lattice formation of WCs **(A,E)**. Anti-DDCA immunofluorescence is shown in green **(B,F)** with nuclei counterstained with DAPI in blue **(C,G)**. Green and blue channels are merged and overlaid with the respective DIC images **(D,H)**. Arrowheads indicate DDCA-immunostaining at the apical membrane of the epithelial cells surrounding the WCs. HL, hemolymph. Scale bar: 20 μm. Reproducible results were obtained from four individual clams for each experimental condition.

**Figure 7:**
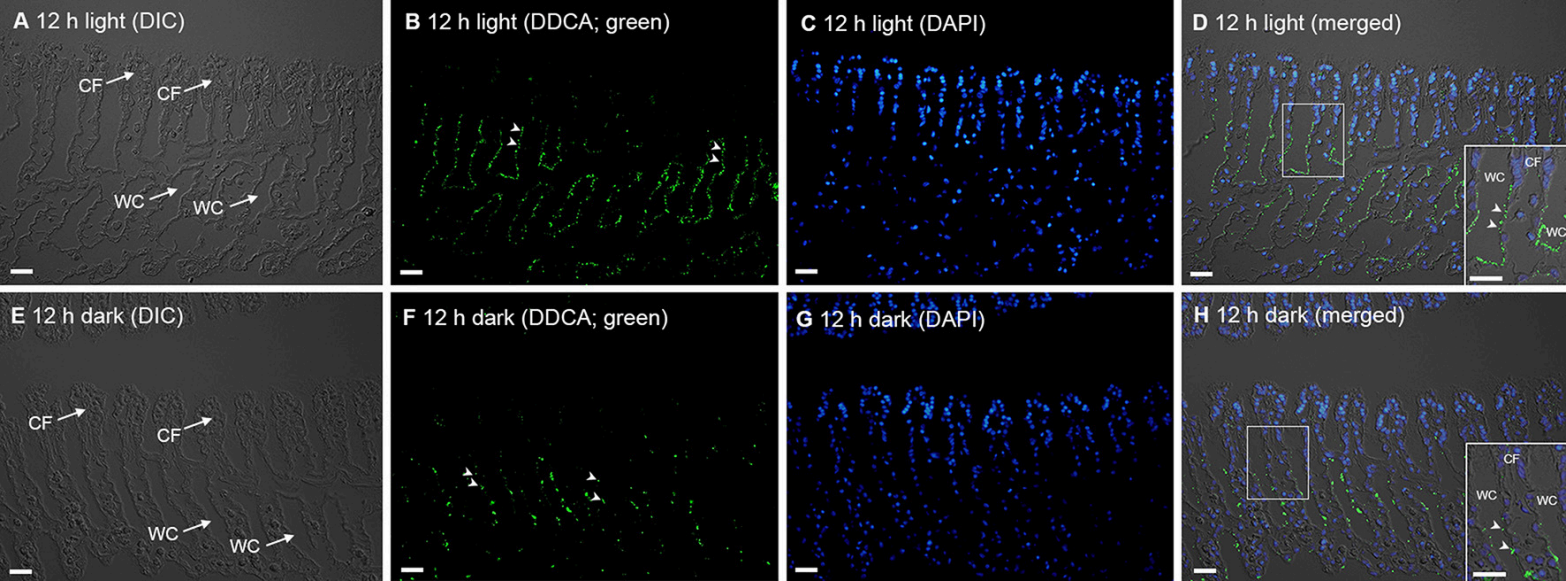
Immunofluorescence localization of the Dual Domain Carbonic Anhydrase (DDCA) in the ctenidial filaments (CFs) of the ctenidium of *Tridacna squamosa*. Immunofluorescence localization of the DDCA in the CFs of *T. squamosa* exposed to 12 h of light **(A–D)** or 12 h of darkness (control; **E–H**). Differential interference contrast (DIC) images show the structure of CFs **(A,E)**. Anti-DDCA immunofluorescence is shown in green **(B,F)** with nuclei counterstained with DAPI in blue **(C,G)**. Green and blue channels are merged and overlaid with the respective DIC images **(D,H)**. Arrowheads indicate DDCA-immunostaining at the apical membrane of the epithelial cells located at the base of CFs and those surrounding the tertiary water channels (WCs). Scale bar: 20 μm. Reproducible results were obtained from four individual clams for each experimental condition.

**Figure 8:**
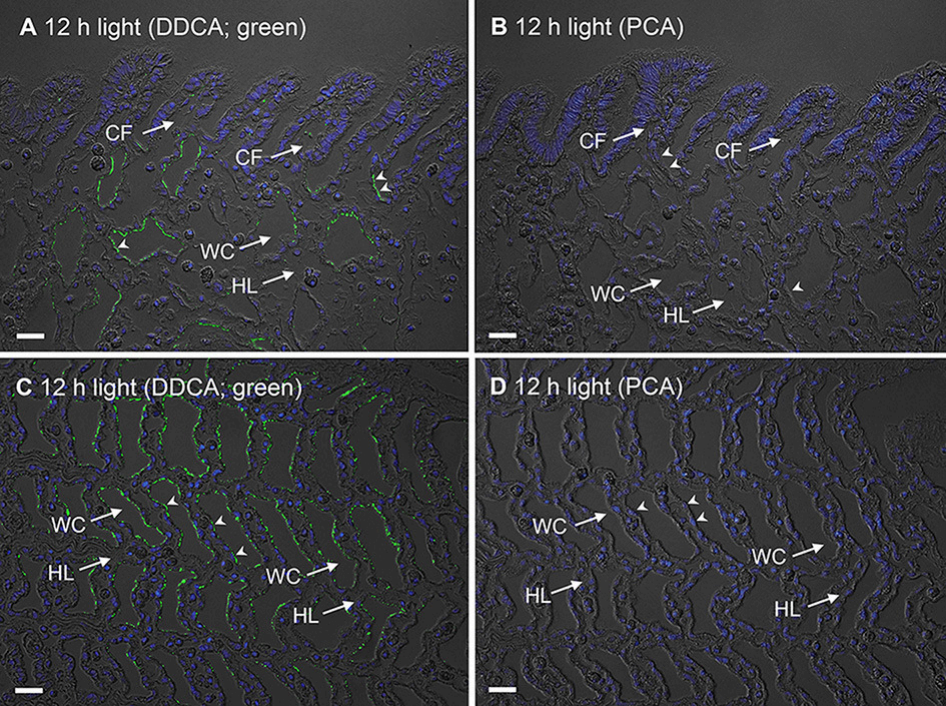
A validation of the immunostaining of the Dual Domain Carbonic Anhydrase (DDCA) using a peptide competition assay (PCA). The immunofluorescence localization of DDCA in the ctenidial filaments (CFs) and tertiary water channels (WCs) of a ctenidium of *Tridacna squamosa* exposed to 12 h of light using an anti-DDCA antibody **(A,C)**, or the same anti-DDCA antibody pre-incubated with the immunizing peptide in PCA **(B,D)**. The anti-DDCA immunofluorescence is shown in green, overlaid with DAPI nuclei staining and differential interference contrast images. Arrowheads in **(A,C)** indicate DDCA-immunostaining at the apical membrane of some epithelial cells at the base of CFs and WCs. By contrast, there is a lack of anti-DDCA antibody staining in the control with PCA **(B,D)** in both CFs and WCs. HL, hemolymph. Scale bar: 20 μm.

In *T. squamosa*, the DDCA-immunofluoresence was detected predominantly at the apical membrane of some epithelial cells surrounding the tertiary water channels (Figure 6) and to a lesser extent at the apical membrane of some epithelial cells at the base of the ctenidial filament (Figure 7). Results obtained through a peptide competition test validated the DDCA-immunostaining (Figure 8). In corroboration with the Western blotting results, more cells surrounding the tertiary water channels of clams exposed to 12 h of light (*N* = 4) displayed apical immunofluorescence epithelial cells as compared with the control kept in darkness for 12 h (*N* = 4).

## Discussion

There are indications that CAs play an important role in alga-invertebrate symbioses. Cnidarians containing endosymbiotic zooxanthellae have significantly higher CA activity (>29 times) than azooxanthellate species (Weis and Reynolds, 1999). The administration of CA inhibitors (acetazolamide and ethoxyzolamide) greatly reduces photosynthetic rates in symbiotic corals and sea anemones, demonstrating the importance of CA to the photosynthetic productivity of zooxanthellae (Al-Moghrabi et al., 1996; Weis and Reynolds, 1999). In addition, CA can be involved in skeleton formation in scleractinian corals by catalyzing ${\text{HCO}}_{3}^{-}$ formation within the sub-calicoblastic space (Tambutté et al., 2007; Moya et al., 2008). In this study, we demonstrated that the DDCA was expressed almost exclusively in the ctenidium of *T. squamosa*, implying that it might have a specialized function unrelated to photosynthesis of its symbionts which are concentrated in the colorful outer mantle, and unassociated with shell formation which involves the whitish inner mantle. We discovered that the DDCA from the ctenidium of *T. squamosa* comprised two separate Zn^2+^-containing active sites, one of which could be positioned extracellularly in seawater while the other one could be located intracellularly in the cytosol. In addition, the gene and protein expression levels of the DDCA in the ctenidium were enhanced by light exposure, corroborating the proposition that it may take part in increased C_i_ uptake from the external medium during insolation.

### The DDCA from *T. squamosa* could be an integral protein with one extracellular and one intracellular Zn^2+^-containing catalytic sites

The DDCA from the ctenidium of *T. squamosa* comprised two distinct α-CA domains (residues 43–285 and residues 315–564), and each of them contained the active site essential for CA catalytic activity as well as the histidine proton shuttle residues (Lindskog, 1997). Hence, both α-CA domains in the DDCA are potentially functional.

A phenogramic analysis revealed the grouping of the DDCA of *T. squamosa* with extracellular CAs of human. This is consistent with immunolocalization of the tgCA as a membrane-associated protein in *T. gigas* (Baillie and Yellowlees, 1998; Leggat et al., 2002). A putative GPI anchor residue was conserved at Gly577 in the tgCA of *T. gigas* (Leggat et al., 2005; or Val575 using the PredGPI predictor program) and Ala576 in the DDCA of *T. squamosa*, suggesting that they could be GPI-anchored proteins. However, Baillie and Yellowlees (1998) demonstrated that digestion with phosphoinositol phospholipase C did not release tgCA from the crude homogenate of ctenidium, denoting that it was probably an integral membrane protein with one or more TM(s) besides being GPI-anchored. Indeed, using a more advanced transmembrane prediction software (Reddy et al., 2014), we demonstrated for the first time the presence of two TMs in the DDCA of *T. squamosa*, one (residue 156–176) in the first α-CA domain near the 5'-end, and another one (residues 580–600) near the ending of the second α-CA domain close to the 3'-end. As the two active sites happened to be separated by a TM, one of them would probably be exposed to the external medium while the other one would be positioned in the cytoplasm. A similar analysis indicated that these two TMs were also present in the tgCA of *T. gigas* (residues 156–176 and residues 579–599, respectively), although no attempt had been made to identify them previously (Leggat et al., 2005).

The CA active site is located in a large conical cavity with a catalytic Zn^2+^ at its bottom (Lindskog, 1997). The Zn^2+^ is held in place by tetrahedral coordination with three His residues and a water molecule or hydroxide ion (Håkansson et al., 1992; Christianson and Fierke, 1996). In the DDCA of *T. squamosa*, the three His residues (His116, His118, His141) of the first α-CA domain were located in front of the TM closer to the 5′ end, denoting that they would be exposed to the external medium. Furthermore, three of the five hydrophilic residues (Asn85, His87, Gln114) of the active site which bind to ${\text{HCO}}_{3}^{-}$ and H^+^, were also located in front on the TM, while four out of the five hydrophobic residues that make up the CO_2_ binding pocket were positioned behind the TM. Taken together, it can be deduced for the DDCA of *T. squamosa* that the active site of the first α-CA domain probably functions in the external medium, binding with exogenous ${\text{HCO}}_{3}^{-}$ and H^+^, catalyzing the dehydration of ${\text{HCO}}_{3}^{-}$ to CO_2_, and releasing the CO_2_ to the cytoplasm. By contrast, the active site of the second α-CA domain is positioned in the cytoplasm and would therefore function intracellularly. With the 3′-end of the DDCA attached to the plasma membrane through another TM and a GPI anchor, the second α-CA domain could function in close proximity to the inner surface of the plasma membrane to catalyze the hydration of CO_2_ that has entered the cell to ${\text{HCO}}_{3}^{-}$. With the two catalytic domains working in close proximity across the two sides of the plasma membrane, the DDCA was configured to facilitate the uptake of exogenous CO_2_ and the assimilation of CO_2_ into ${\text{HCO}}_{3}^{-}$ in the ctenidial epithelial cells of *T. squamosa*.

### The evolutionary trend of CAs with two catalytic domains

Besides the DDCA of *T. squamosa* and the tgCA of *T. gigas*, only four other CAs are known to contain two domains. The deep-sea bivalve *Phreagena okutanii* (Hongo et al., 2016) and the green alga *D. salina* (Fisher et al., 1996) express CAs that comprise two α-CA domains, while the bacterium *Halothiobacillus neapolitanus* (Sawaya et al., 2006) and the red alga *P. purpureum* have CAs that consist of two β-CA domains (Mitsuhashi and Miyachi, 1996).

β-CAs are a diverse but structurally related group of CAs found in eubacteria, plant chloroplasts, red and green algae, and the Archaea (Rowlett, 2010). They can adopt a variety of oligomeric states with molecular masses ranging from 45 to 200 kDa, and the basic structural unit is a dimer or its structural equivalent. The basic dimer consists of two identical protein chains of 25–30 kDa, each with a catalytic domain of 100% similarity. The β-CA of *P. purpureum* may act as a CO_2_-concentrating mechanism to maintain a favorable *P*CO_2_ in order to activate the RuBisCO-catalyzed photosynthesis. In *P. purpureum*, the β-CA monomer consists of 571 amino acids with a molecular mass of 55 kDa (Mitsuhashi and Miyachi, 1996); the monomer comprises two catalytic domains with two atoms of zinc, each equivalent to the catalytic domains of other β-CAs (25–30 kDa). These two domains are arranged in tandem and exhibit ~70% identity with each other. It has been suggested that the β*-CA* gene of *P. purpureum* is formed by the duplication and fusion of a primordial β*-CA* gene (Mitsuhashi et al., 2000). On the other hand, CsoSCA is a bacterial carbonic anhydrase localized in the boundary of a cellular micro-compartment called the carboxysome, where it also converts ${\text{HCO}}_{3}^{-}$ to CO_2_ for use in carbon fixation by RuBisCO. While CsoSCA of *H. neapolitanus* contains a pair of fused and homologous domains, the two catalytic domains have diverged (with only ~11% similarity) to the point that only one domain in the pair retains a functional active site (Sawaya et al., 2006). Hence, it would appear that the evolution of dual domain β-CAs among different groups of organisms involved disparate changes in the active sites of the two catalytic domains.

In comparison, the DDCA of *D. salina* comprises two α-CA domains, probably also derived from an internal duplication and concatenation (Fisher et al., 1996). The two catalytic domains have 70% sequence similarity, indicating that they had diverged after gene duplication and concatenation, similar to the dual domain β-CA of *P. purpureum*. Notably, the DDCA of *D. salina* is largely hydrophilic and does not contain any TM (Fisher et al., 1996). As for the DDCA of *P. okutanii* (MCACO2; Hongo et al., 2016), it consists of 594 amino acids with two α-CA domains, which share only 37% sequence similarity. Its C-terminus comprises a TM of 15 amino acids (residues 580–594) and a possible GPI anchor residue (Ser570). In the ctenidium of *P. okutanii*, the MCACO2 is postulated to be anchored at the 3' end to the apical membrane of the asymbiotic non-ciliated cells, and the two catalytic sites are apparently positioned in the external medium to facilitate the uptake of CO_2_ from seawater in support of carbon fixation in the thioautotrophic bacterial symbionts (Hongo et al., 2016). With the discovery of two TMs in the DDCA of *T. squamosa*, and one of them separating the two active sites in particular, it becomes apparent that the physiological functions of DDCAs can be modified during evolution not only through variations in the amino acid compositions of the two catalytic domains, but also by the acquisition of TMs to anchor the protein, or to position the two active sites in two separate compartments across the plasma membrane, or both.

### The DDCA of *T. squamosa* is localized to the apical membrane of the epithelial cells in the ctenidium

Similar to the gills of fishes, the ctenidia of giant clams are highly convoluted structures specialized for the exchange of gases and small molecules between the hemolymph and the external medium. The major anatomical features of the ctenidium are finger-like filaments, ciliated water channels, haemal sinuses that contain hemolymph, as well as a few tertiary tubules containing zooxanthellae (Leggat et al., 2002). In *T. gigas*, the tgCA is found in the ciliated cuboidal epithelium lining the water channels of the ctenidium (Baillie and Yellowlees, 1998). In the case of *T. squamosa*, we report for the first time the subcellular localization of DDCA in the apical membrane of the epithelium cells of the ctenidial water channels, which provides clues to its possible physiological functions.

In non-symbiotic marine organisms, metabolic CO_2_ as a waste product is excreted mainly through the gills. For example, a membrane-associated CA has been localized to the basolateral membrane of the branchial epithelial cells in crabs (Burnett and McMahon, 1985; Henry et al., 2003). With such a location, the catalytic CA domain is in direct contact with the hemolymph and therefore catalyzes the dehydration of ${\text{HCO}}_{3}^{-}$ therein into endogenous CO_2_, which can then diffuse out to the seawater for excretion (Burnett, 1984; Burnett et al., 1985). In contrast, symbiotic organisms like giant clams need to acquire C_i_ from the seawater. Therefore, the apical localization of DDCA in the ctenidial epithelial cells surrounding the tertiary water channels suggests that it would catalyze the conversion of ${\text{HCO}}_{3}^{-}$ in the seawater to CO_2_, and facilitate the diffusion of exogenous CO_2_ through the epithelial cells of the water channels into the hemolymph.

### Light enhances the expression of *DDCA*/DDCA in the ctenidium and its implications

In algae and plants, the activity and/or expression of CAs can be changed by light (Majeau and Coleman, 1996; Moskvin et al., 2000) or UV radiations (Wu and Gao, 2009). By contrast, the activity and/or expression of CAs in animals are not known to be light-responsive, although they can be affected by CO_2_ (oyster; Wang et al., 2017), salinity stress (crab; Henry et al., 2006; Serrano et al., 2007), temperature (fish; Houston and Mearow, 1979), copper and osmotic stress (fish; de Polo et al., 2014), or hypoxia (human cancer cells; Ambrosio et al., 2016). In this study, we have demonstrated that light can augment the transcript level and protein abundance of the *DDCA*/DDCA in the ctenidium of *T. squamosa*, with the former (at hour 6) preceding the latter (at hour 12), indicating that the DDCA is regulated at both the transcriptional and translational levels. The increase in the protein abundance of the ctenidial DDCA after 12 h of light exposure was apparently not a circadian phenomenon, because such an increase was absent from the parallel controls exposed to darkness for a total of 24 h. However, whether circadian rhythm plays a role in regulating the expression levels of other genes and proteins in various tissues/organs of *T. squamosa* deserves a more detailed investigation in the future. Because of the daily light:dark cycle, changes in transcript levels and protein abundance of the *DDCA*/DDCA would logically be short-term and relatively moderate as compared with changes in other types of animal CAs in response to long-term environmental changes (e.g., salinity changes in crabs; Henry et al., 2006; Serrano et al., 2007).

The increased expression of DDCA during light exposure would theoretically catalyze a greater rate of CO_2_ formation from ${\text{HCO}}_{3}^{-}$ around the water channels and at the base of the filaments. However, the increased dehydration of ${\text{HCO}}_{3}^{-}$ requires a concurrently increase in the excretion of H^+^ to the external medium. As expected, the transcript level and protein abundance of the NHE3-like transporter are up-regulated in the ctenidium of *T. squamosa* exposed to light, probably to augment H^+^ excretion in the pursuance of whole-body acid-base balance (Hiong et al., 2017b). Concomitantly, the excreted H^+^ can react with the ${\text{HCO}}_{3}^{-}$ in the external medium, catalyzed by the active site of the first CA domain of DDCA, releasing the CO_2_ formed to the cell interior. Once inside the epithelial cells, the absorbed CO_2_ can be hydrated back to ${\text{HCO}}_{3}^{-}$ catalyzed by the active site of the second CA domain of DDCA. Then, ${\text{HCO}}_{3}^{-}$ can exit the basolateral membrane via some sort of ${\text{HCO}}_{3}^{-}$ transporters and pass into the hemolymph. Taken altogether, it is logical to conclude that the DDCA, with its light-dependent gene and protein expression, could augment the uptake of exogenous C_i_ through the ctenidium of *T. squamosa* during insolation.

### The involvement of other CAs and transporters to deliver the absorbed C_i_ to the symbionts

It has been reported previously that zooxanthellae isolated from *T. gigas* possess a carbon-concentrating mechanism; they can utilize CO_2_ and ${\text{HCO}}_{3}^{-}$ from the ambient seawater and accumulate C_i_ intracellularly (Leggat et al., 1999). Zooxanthellal CA can be part of the carbon-concentration mechanism as the isolated zooxanthellae display light-enhanced CA activity (Leggat et al., 1999). At present, it remains unknown whether light affects the transcript and protein expression levels of a specific isoform of cytosolic CA in these isolated zooxanthellae. However, Ip et al. (2017b) has recently cloned a *CA2* homolog (*CA2-like*) of host-origin from the fleshy and colorful outer mantle of *T. squamosa*. CA2-like is localized to the tubule epithelial cells, and light enhances its protein abundance significantly in the outer mantle. Hence, CA2-like could probably take part in the increased supply of C_i_ from the host clam to the photosynthesizing symbiotic zooxanthellae during insolation. Unlike symbiotic cnidarians, giant clams have distinct tissues and organs with high degree of division of labor between them. Therefore, in the case of *T. squamosa*, it is unsurprising that the uptake of exogenous C_i_ and its delivery to the zooxanthellae by the host clam require the cooperation between the epithelial cells of the ctenidium and those of the zooxanthellal tubules with the involvement of different types of host CAs. It is probable that some other CA isoforms yet to be identified are also involved in these processes.

### Perspectives on light-dependent expression of enzymes/transporters in *T. squamosa*

The hemolymph of giant clams has a pH of 7.4–7.6 (*Tridacna maxima*, Deane and O'Brien, 1980; *T. gigas*, Fitt et al., 1995). In *T. gigas*, the concentration of C_i_, present mainly as ${\text{HCO}}_{3}^{-}$, in the hemolymph ranges between 1.8 and 2.2 mmol l^−1^ (Yellowlees et al., 1993). During insolation, the concentration of C_i_ in the hemolymph of *T. gigas* decreases to 1.6 mmol l^−1^ (Yellowlees et al., 1993), while the pH of the hemolymph increases by 0.5 unit, resulting mainly from the decrease in inorganic carbon concentration (Fitt et al., 1995). This is logical as light induces photosynthesis in the symbiotic zooxanthellae, and photosynthesis would lower the ${\text{HCO}}_{3}^{-}$ concentration in and simultaneously increase the pH of the hemolymph in the host clam. Therefore, the possibility of the transcript and protein levels of *DDCA*/DDCA in the ctenidium of *T. squamosa* changing in response to the hemolymph ${\text{HCO}}_{3}^{-}$ concentration (i.e., indirectly to light) cannot be ignored. However, other enzymes and transporters, some of which unrelated to ${\text{HCO}}_{3}^{-}$, also display light-enhanced gene and/or protein expression in *T. squamosa*. These include the Glutamine Synthetase (Hiong et al., 2017a), the NHE3-like (Hiong et al., 2017b), and the urea active transporter DUR3-like (Chan et al., 2018) of the ctenidium as well as the Plasma Membrane Ca^2+^-ATPase (Ip et al., 2017a) and the Na^+^/K^+^-ATPase α-subunit (Boo et al., 2017) of the inner mantle. Hence, it is logical to conclude that *T. squamosa* may have developed a general mechanism to coordinate the expression levels of a variety of enzymes and transporters in relation to various diurnally light-dependent physiological processes, including light-enhanced C_i_ absorption, light-enhanced ammonia absorption and assimilation, and light-enhanced calcification. This could be a result of the symbiotic relationship between the host clam and the symbiotic zooxanthellae, as the host would also need to be light-responsive in order to support the light-induced photosynthesis in its symbionts.

Zooxanthellae can respond to light directly probably because they possess a photoreceptor protein, opsin, and eye-spots made of crystalline clusters of uric acid (Yamashita et al., 2009). However, the mechanism of light-dependent changes in gene and protein expression in the host clam is intriguing, because the ctenidium lacks pigment, and animal tissues without pigments are usually not light-responsive. As proposed previously (Ip et al., 2015; Hiong et al., 2017a), it is possible that the symbionts produce some sort of signaling molecules in response to light, and when these signaling molecules are released to the extracellular fluid of the host clam, they can activate the transcription and/or translation of *DDCA*/DDCA and other enzymes/transporters in the host cells. Another possibility is that light is sensed through the host's siphonal eyes (Wilkens, 1986), which then transmit neural or chemical signals to other parts of the body. Hence, efforts should be made in the future to elucidate the signaling mechanisms pertaining to the light-enhanced expression of genes and proteins in *T. squamosa*.

Transcriptional and translational processes entail energy expenditure, and therefore the diel cycle of changes in gene and protein expressions in *T. squamosa* would appear to be energetically uneconomical. However, unlike free-living animals, giant clams receive a continuous supply of nutrients from the symbiotic zooxanthellae, including glycerol, glucose, and amino acids (Muscatine, 1967; Muscatine et al., 1983; Edmunds and Davies, 1986; Streamer et al., 1988; Davies, 1991). The quantity of translocated nutrients is sufficient to meet the daily energy and growth requirements of the host clam (Fisher et al., 1985; Klumpp et al., 1992; Klumpp and Griffith, 1994; Hawkins and Klumpp, 1995). Probably because of that, *T. squamosa* could afford energetically to regulate light-inducible processes through light-dependent diurnal changes in transcription and translation. Such diurnal changes would imply that the related transcripts and proteins must undergo rapid turnover, and therefore it would be important to determine the half-life of these proteins and to examine the regulatory mechanisms of the turnover process in the future.

## Author contributions

YI designed the study and drafted the manuscript. CK, KH, CC, MB, and WW performed the experiment. CK, KH, CC, MB, SC, MN, and YI analyzed the data. WW and SC contributed reagents, materials and analysis tools. All authors reviewed the manuscript.

### Conflict of interest statement

The authors declare that the research was conducted in the absence of any commercial or financial relationships that could be construed as a potential conflict of interest.
