# Supplementary material for: Molecular Characterization of a Dual Domain Carbonic Anhydrase From the Ctenidium of the Giant Clam, Tridacna squamosa, and Its Expression Levels After Light Exposure, Cellular Localization, and Possible Role in the Uptake of Exogenous Inorganic Carbon
Source: Front Physiol. 2018 Mar 26;9:281. doi: 10.3389/fphys.2018.00281 (PMC5879104; doi:10.3389/fphys.2018.00281)
Supplement: Supplementary file 1 [file Table1.DOCX]

| No | Description | Accession number | E-value | Max score (bits) |
| --- | --- | --- | --- | --- |
| 1 | Carbonic anhydrase precursor [*Tridacna gigas*] | AAX16122.1 | 9e-142 | 419 |
| 2 | Putative two domain conserved membrane-associated carbonic anhydrase [*Phreagena okutanii*] | BAU71500.1 | 2e-61 | 211 |
| 3 | PREDICTED: carbonic anhydrase 2 [*Crassostrea gigas*] | XP_011434938.1 | 1e-55 | 189 |
| 4 | PREDICTED: carbonic anhydrase-like [*Aplysia californica*] | XP_012935478.1 | 3e-55 | 195 |
| 5 | PREDICTED: carbonic anhydrase 15-like [*Jaculus jaculus*] | XP_004670914.1 | 3e-54 | 186 |
| 6 | Carbonic anhydrase 9 [*Stegodyphus mimosarum*] | KFM74434.1 | 2e-53 | 182 |
| 7 | PREDICTED: carbonic anhydrase 1-like [*Crassostrea gigas*] | XP_011455036.1 | 2e-53 | 183 |
| 8 | Hypothetical protein LOTGIDRAFT_239341 [*Lottia gigantea*] | XP_009053021.1 | 3e-53 | 182 |
| 9 | PREDICTED: carbonic anhydrase 15-like [*Equus przewalskii*] | XP_008507980.1 | 1e-52 | 182 |
| 10 | PREDICTED: carbonic anhydrase 4-like [*Cynoglossus semilaevis*] | XP_008307494.1 | 4e-52 | 180 |

**Table S1**. The top 10 results from a protein BLAST (BlastP program, version 2.6.0) of the deduced amino acid sequence of the first CA domain of DDCA (residue 43-285) from the ctenidia of *Tridacna squamosa* using default settings.
