# Supplementary material for: Molecular Characterization of a Dual Domain Carbonic Anhydrase From the Ctenidium of the Giant Clam, Tridacna squamosa, and Its Expression Levels After Light Exposure, Cellular Localization, and Possible Role in the Uptake of Exogenous Inorganic Carbon
Source: Front Physiol. 2018 Mar 26;9:281. doi: 10.3389/fphys.2018.00281 (PMC5879104; doi:10.3389/fphys.2018.00281)
Supplement: Supplementary file 2 [file Table2.DOCX]

| No | Description | Accession number | E-value | Max score (bits) |
| --- | --- | --- | --- | --- |
| 1 | Carbonic anhydrase precursor [*Tridacna gigas*] | AAX16122.1 | 3e-160 | 466 |
| 2 | Putative two domain conserved membrane-associated carbonic anhydrase [*Phreagena okutanii*] | BAU71500.1 | 9e-79 | 257 |
| 3 | Carbonic anhydrase-like [*Mizuhopecten yessoensis*] | XP_021340717.1 | 4e-55 | 194 |
| 4 | Carbonic anhydrase [*Tridacna gigas*] | AAY82323.1 | 3e-54 | 180 |
| 5 | PREDICTED: carbonic anhydrase 4-like [*Poecilia latipinna*] | XP_014896624.1 | 7e-52 | 180 |
| 6 | PREDICTED: carbonic anhydrase 4-like [*Poecilia mexicana*] | XP_014828708.1 | 3e-51 | 179 |
| 7 | PREDICTED: carbonic anhydrase 4-like [*Poecilia formosa*] | XP_007567395.1 | 3e-51 | 179 |
| 8 | PREDICTED: carbonic anhydrase 2 [*Crassostrea gigas*] | XP_011434938.1 | 4e-51 | 178 |
| 9 | Carbonic anhydrase-like [*Mizuhopecten yessoensis*] | XP_021373119.1 | 4e-51 | 178 |
| 10 | PREDICTED: carbonic anhydrase 4-like [*Xiphophorus maculatus*] | XP_005811029.2 | 5e-51 | 177 |

**Table S2**. The top 10 results from a protein BLAST (BlastP program, version 2.6.0) of the deduced amino acid sequence of the second CA domain of DDCA (residue 315-564) from the ctenidia of *Tridacna squamosa* using default settings.
